# Corresponding myxoid liposarcoma diagnosed on fine needle aspiration cytology: more to it than meets the eye

**DOI:** 10.1590/1806-9282.20250698

**Published:** 2026-04-20

**Authors:** Demet Sengul, Ilker Sengul

**Affiliations:** 1 Giresun University, Faculty of Medicine, Department of Pathology – Giresun, Turkey.; 2 Giresun University, Faculty of Medicine, Division of Endocrine Surgery – Giresun, Turkey.; 3 Giresun University, Faculty of Medicine, Department of General Surgery – Giresun, Turkey.

Dear Editor,

Sarcoma, per se, remains a dynamic and challenging issue for healthcare providers to date^1–3^. We read with a great deal of interest the article entitled "Myxoid liposarcoma diagnosed on fine needle aspiration cytology: There is more to it than meets the eye" by Osama et al^4^. This article gives a valuable contribution to the literature by highlighting the cytological features of a rare mesenchymal neoplasm, myxoid liposarcoma (MLPS). The authors effectively emphasize the importance of fine needle aspiration (FNA) cytology as a minimally invasive and rapid method for the initial assessment of lipomatous lesions. The case presentation of MLPS in the uncommon location of the calf in a 19-year-old boy further adds to the significance of this report, as MLPS typically has a predilection for the lower extremities, particularly the thigh. As the authors correctly point out, occurrences in the calf are pretty rare. Of note is that the study successfully purposed to improve the understanding of MLPS and hinder misdiagnosis by evaluating cytology specimens. The inclusion of detailed descriptions of the cytological features, such as clusters of spindled to stellate cells embedded in an abundant myxoid matrix, the characteristic "chicken wire pattern" of delicate thin-walled capillaries, and the occasional presence of small, multivacuolated lipoblasts with nuclear scalloping, is particularly beneficial. The correlation of these cytological findings with the subsequent histopathological examination reinforces the accuracy of FNA in diagnosing MLPS in this context. Furthermore, the discussion section appropriately addresses the differential diagnosis of myxoid adipocytic tumors, including intramuscular myxoma, myxoid neurogenic tumors, and the myxoid variant of malignant fibrous histiocytoma.

While this report provides insightful information, it also has some flaws. For instance, the inherent limitation of being a single case study should be acknowledged, as it may limit the generalizability of the findings to a broader population. Additionally, although the characteristic morphology allowed for diagnosis without immunohistochemistry in this specific case, the inclusion of immunohistochemical analysis in future reports could provide further validation and assist in diagnostically challenging cases. In fine, given the importance of imaging in MLPS diagnosis, a more in-depth discussion of the variability of magnetic resonance imaging findings and their correlation with histological features could further enhance the understanding of this tumor entity, as alluded to in the debate. Despite these points, this case report by Osama et al.^4^ is a valuable contribution to the literature on myxoid liposarcoma. This publication serves as a helpful resource for pathologists, especially those with limited experience in evaluating soft tissue sarcomas, and underscores the importance of a multidisciplinary approach for improved long-term survival and patient outcomes in MLPS for physicians. This issue merits further investigation. We thank Osama et al.^4^ for their study.

## Data Availability

The datasets generated and/or analyzed during the current study are available from the corresponding author upon reasonable request.
